# Phenotypic and Genotypic Comparison of a Live-Attenuated Genotype I Japanese Encephalitis Virus SD12-F120 Strain with Its Virulent Parental SD12 Strain

**DOI:** 10.3390/v12050552

**Published:** 2020-05-16

**Authors:** Muhammad Naveed Anwar, Xin Wang, Muddassar Hameed, Abdul Wahaab, Chenxi Li, Mona Sharma, Linlin Pang, Muhammad Irfan Malik, Ke Liu, Beibei Li, Yafeng Qiu, Jianchao Wei, Zhiyong Ma

**Affiliations:** Shanghai Veterinary Research Institute, Chinese Academy of Agricultural Sciences, Shanghai 200241, China; dr.naveed903@gmail.com (M.N.A.); wang1655609668@outlook.com (X.W.); mudasar386@gmail.com (M.H.); abdul.wahaab@uaf.edu.pk (A.W.); lichenxihsy@outlook.com (C.L.); monasharma1990@yahoo.com (M.S.); PLL20200506@outlook.com (L.P.); iffiathangal@yahoo.com (M.I.M.); liuke@shvri.ac.cn (K.L.); lbb@shvri.ac.cn (B.L.); yafengq@shvri.ac.cn (Y.Q.)

**Keywords:** Japanese encephalitis virus, attenuation, virulence, vaccine, genotype, amino acid substitution

## Abstract

The phenotypic and genotypic characteristics of a live-attenuated genotype I (GI) strain (SD12-F120) of Japanese encephalitis virus (JEV) were compared with its virulent parental SD12 strain to gain an insight into the genetic changes acquired during the attenuation process. SD12-F120 formed smaller plaque on BHK-21 cells and showed reduced replication in mouse brains compared with SD12. Mice inoculated with SD12-F120 via either intraperitoneal or intracerebral route showed no clinical symptoms, indicating a highly attenuated phenotype in terms of both neuroinvasiveness and neurovirulence. SD12-F120 harbored 29 nucleotide variations compared with SD12, of which 20 were considered silent nucleotide mutations, while nine resulted in eight amino acid substitutions. Comparison of the amino acid variations of SD12-F120 vs. SD12 pair with those from other four isogenic pairs of the attenuated and their virulent parental strains revealed that the variations at E^138^ and E^176^ positions of E protein were identified in four and three pairs, respectively, while the remaining amino acid variations were almost unique to their respective strain pairs. These observations suggest that the genetic changes acquired during the attenuation process were likely to be strain-specific and that the mechanisms associated with JEV attenuation/virulence are complicated.

## 1. Introduction

Japanese encephalitis (JE) caused by Japanese encephalitis virus (JEV) is endemic in Asia-Pacific regions including the world’s most populous countries: China, India, Indonesia, Pakistan, and Bangladesh. JE is a leading cause of encephalitis and its incidence is expected to increase in some countries [1,2]. JEV infection typically results after 5–15 days with a non-specific febrile illness, accompanied by headache, vomiting, reduced consciousness, and convulsions [3]. JEV is a member of the genus *Flavivirus* in the family *Flaviviridae* has a single-strand, positive-sense RNA genome that is nearly 11 kb in length [4]. Structurally, JEV genomic RNA contains an approximately 10,296 nucleotide (nt) coding region flanked by the 5ʹ and 3ʹ untranslated regions (UTR) that comprises an array of regulatory RNA elements required for viral genome replication and translation [5,6]. The JEV coding region encodes a polyprotein precursor that is processed after translation by viral and/or host cellular proteases into ten discrete products: [7] three structural proteins (capsid (C), precursor membrane (prM), and envelope (E)) and seven non-structural proteins (NS1, NS2A, NS2B, NS3, NS4A, NS4B, and NS5). The structural proteins are essential for formation of infectious viral particles, while the non-structural proteins are involved in viral RNA replication, viral particle assembly, and evasion of innate immunity [8,9].

JEV is phylogenetically classified into five genotypes (GI to GV) based on the nucleotide sequence of the viral envelope (E) gene [3,10]. GIII was isolated in Japan in 1935 and was the dominant genotype until the end of the 20th century in most countries in Asia [11,12]. GI was first identified in Cambodia in 1967 and was not detected until a new strain re-emerged in China in 1979 [13,14]. Previous surveillance data have suggested that the number of GI isolates has been increasing in the past 20 years, thus resulting in a JEV genotype shift from GIII to GI in many Asian countries [15]. GI strains replicate more efficiently than GIII strains in JEV amplifying hosts, which has been considered to play a role in the JEV genotype shift [16,17]. GI strains harbor a variety of amino acid substitutions, of which NS2B-V99L/NS3-A78S/E177E substitutions have recently been demonstrated to contribute to the replication advantage of GI strains over GIII strains in pigs and poultry [17].

JEV is a vaccine-preventable pathogen. Currently, four different types of JE vaccines: the mouse brain-derived killed-inactivated Nakayama vaccine, the cell culture-derived live-attenuated SA14-14-2 vaccine (SA14-14-2 vaccine), the culture-derived killed-inactivated vaccine, and the genetically engineered live-attenuated chimeric vaccine, are available for humans in various areas of the world [18], all of which are derived from GIII strains. The mouse brain-derived killed-inactivated Nakayama vaccine is no longer used and other vaccines have taken its place. SA14-14-2 vaccine is extensively used in many Asian countries; however, the protective efficacies of GIII-derived vaccines against G1 infection in mice are not consistent [19,20]. GI viruses have been isolated from patients vaccinated with SA14-14-2 vaccine in China and India [21,22]. These observations have raised a concern about the potential need for a GI-derived vaccine [23]. Several GI vaccines are being developed [24,25].

The most commonly used JEV vaccine strain in the vaccine industry is SA14-14-2, which was derived from its virulent parental SA14 strain through an intricate course of ~150 serial passages in cultured cells and non-neural tissues/organs of live animals [26]. During the serial passages, the spontaneous substitutions of amino acids resulted in a loss of JEV virulence. JEV virulence is defined by two properties: (i) neuroinvasiveness, that is the ability of JEV to enter the central nervous system (CNS) when inoculated by a peripheral route; and (ii) neurovirulence, that is the ability of JEV to replicate and cause damage within the CNS when inoculated directly into the brain of a host [27]. More than 17 amino acid substitutions present in viral E and other proteins are acquired during the attenuation process of SA14 [28]. Some substitutions at E^107^, E^138^, E^176/177^, and E^279^ of E protein differentially contribute to the neurovirulence attenuation of SA14-14-2 [29], improving the understanding of the mechanisms associated with SA14-14-2 attenuation. However, the mechanisms associated with JEV attenuation/virulence are complicated and remain largely unknown. We have previously produced a live-attenuated GI strain (SD12-F120) by 120 serial passages of its virulent parental SD12 strain in BHK-21 cells (unpublished data). In the present study, we performed a comparative analysis of the phenotypic and genotypic properties of the isogenic SD12-F120 and SD12 strains, which differed in terms of viral replication, neuroinvasiveness and neurovirulence, and genome sequence.

## 2. Materials and Methods

### 2.1. Ethics Statement

All animal experiments were approved by the Institutional Animal Care and Use Committee of Shanghai Veterinary Research Institute (IACUC No: Shvri-mo-2018110508) and performed in compliance with the Guidelines on the Humane Treatment of Laboratory Animals (Ministry of Science and Technology of the People’s Republic of China, Policy No. 2006 398).

### 2.2. Viruses and Cells

Virulent parental GI SD12 strain was isolated from the brain tissues of an aborted swine fetus [30]. The attenuated JEV SD12-F120 was derived from SD12 by 120 passages in baby hamster kidney cell line (BHK-21) (Supplementary Table S1). BHK-21 cells were maintained in Dulbecco’s modified Eagle’s medium (DMEM) (Thermo Fisher Scientific, Carlsbad, CA, USA) supplemented with 10% fetal bovine serum (FBS) at 37 °C in an atmosphere containing 5% CO_2_. Mouse primary neuron cells were prepared from 24 h newborn baby mice (Shanghai SLAC Laboratory Animal Co. LTD) as described previously [31] and cultured in Neurobasal medium supplemented with 1% B27 vitamin (Thermo Fisher Scientific) and 1% penicillin-streptomycin solution at 37 °C in 5% CO_2_.

### 2.3. Attenuation of SD12 by Serial Passaging in BHK-21 Cells

BHK-21 cell monolayer in T25 flask was inoculated with SD12 at 0.01 MOI. After 1 h adsorption, the cells were washed three times with phosphate-buffered saline (PBS) and cultured in fresh DMEM supplemented with 2% FBS at 37 °C until the appearance of a substantial cytopathic effect. The supernatants were harvested and stored in aliquots at −80 °C for repeated culture. The SD12 underwent serial passages from passage 1 to 120 in BHK-21 cells. Three plaque purifications on BHK-21 cells and virulence tests in three-week-old mice were performed at passage 100 and 120, respectively.

### 2.4. Plaque Size Determination

BHK-21 cells precultured on 6 well plates (3 × 10^5^ cells per well) were mock-inoculated or inoculated with 10-fold serial dilutions of SD12 or SD12-F120 and incubated at 37 °C for 1 h. The cells were washed twice with PBS and overlaid with 4 mL DMEM containing 1% low gelling temperature agarose and 2% FBS. The cells were fixed with 4% paraformaldehyde at 5 days post-infection (dpi) and stained with 0.5% crystal violet. Plaques were visualized and their size was determined.

### 2.5. Neuroinvasiveness and Neurovirulence Tests in Mice

Three-week-old weanling female C57BL/6 mice (10 mice/group) purchased from the Shanghai SLAC Laboratory Animal Co. LTD were inoculated intraperitoneally (100 μL/each) or intracerebrally (30 μL/each) with either SD12 or SD12-F120, respectively, at doses ranging from 10^0^ to 10^6^ plaque-forming unit (PFU) to measure neuroinvasiveness or neurovirulence. The mice were monitored daily for 20 days. Mice that showed neurological signs of seizures, tremors, and paresis were euthanized by CO_2_ asphyxiation, followed by cervical dislocation according to the Guidelines on the Humane Treatment of Laboratory Animals (Policy No. 2006 398). The 50% lethal dose (LD_50_) values were determined by the method of Reed and Muench for both these viruses [32].

### 2.6. Vaccination and Challenge

Three-week-old weanling female C57BL/6 mice (8 mice/group) were mock-vaccinated or vaccinated intraperitoneally with SD12-F120 at doses ranging from 10^2^ to 10^4^ PFU (100 μL/each). After 14 days of vaccination, the mice were challenged intraperitoneally with SD12 at a dose of 10^3^ and 10^4^ PFU (100μL/each) and were monitored daily for 20 days. Mice that showed neurological signs were euthanized according to the Guidelines on the Humane Treatment of Laboratory Animals (Policy No. 2006 398).

### 2.7. Detection of JEV Replication in Mouse Brains

Three-week-old weanling female C57BL/6 mice (10 mice/group) were inoculated intracerebrally with either SD12 or SD12-F120 at a dose of 10^3^ PFU (30 μL/each). Brain tissues were collected at 3, 5, and 7 dpi, and JEV replication was detected by a quantitative real-time reverse transcription-polymerase chain reaction (qRT-PCR) with primer pair and probe (Supplementary Table S2) for amplification of JEV PrM gene as described previously [33].

### 2.8. Detection of Expression of NS1 and NS1’ in BHK-21 Cells

BHK-21 cells infected with either SD12 or SD12-F120 at an MOI of 0.01 were harvested and subjected to western blot analysis of the expression of NS1 and NS1’, as described previously [34]. The polyclonal antibodies against NS1 and NS1’ were generated in our laboratory.

### 2.9. Viral Genome Sequencing

JEV genomic RNA was isolated from the supernatants of JEV infected BHK-21 cells using RNA Mini Kit (TaKaRa, Shiga, Japan) and reverse transcribed into cDNA using PrimeScript^TM^ RT Master Mix (TaKaRa). JEV gene was amplified with four specific primer pairs representing the full-length viral genome using high-fidelity DNA polymerase (New England Biolabs, Ipswich, MA, USA) based on SD12 sequence (GenBank No. MH753127) (Supplementary Table S2). PCR products were excised from an agarose gel and purified with QIAquick Gel Extraction Kit (Qiagen, Valencia, CA, USA). The purified products were bidirectionally sequenced by Sanger sequencing by Invitrogen Corporation (Shanghai, China). The phylogenetic tree was constructed based on the nucleotide sequences of E gene by MEGA version 7.0 software using SD12 and SD12-F120 strains and 25 known JEV strains belonging to different genotypes.

### 2.10. Sequence Analysis

The nucleotide and deduced amino acid sequences of SD12-F120 were compared with SD12. The resulting variations of SD12 vs. SD12-F120 pair were further compared with those from another four isogenic pairs of the attenuated and their virulent parental strains including two GI pairs (SCYA201201-0901 vs. SCYA201201 pair, 10S3 vs. HEN0701 pair) and two GIII pairs (SA14-14-2 vs. SA14 pair, RP-2ms vs. RP-9 pair). The information on the production of each pair was provided in Supplementary Table S1.

### 2.11. Statistical Analysis

All data were processed using Graph Pad Prism 7.0 (GraphPad, La Jolla, CA, USA). Student’s *t*-test was used for statistical analyses. Kaplan-Meier estimates were plotted for the time-to-death data observed in each group and pairwise log-rank tests were used to determine if time to death was significantly different among the groups. A *p* value < 0.05 was considered statistically significant.

## 3. Results

### 3.1. Replication Phenotype and NS1ʹ Expression in BHK-21 Cells

**Figure 1:**
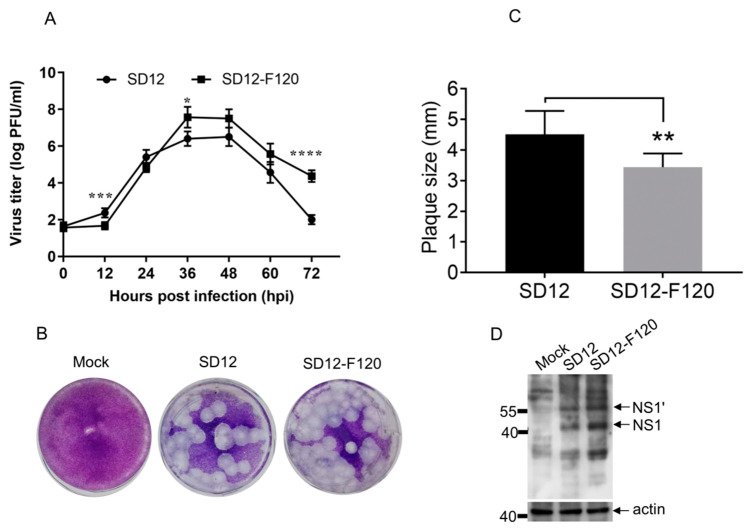
Replication phenotypes of SD12-F120 and SD12 in BHK-21 cells. (**A**) BHK-21 cells were inoculated with SD12-F120 or SD12 at an MOI of 0.01 and the supernatants were collected at the indicated time intervals. JEV titers in the supernatants were measured by plaque assay. (**B**,**C**) BHK-21 cells were mock-infected or infected with SD12-F120 or SD12 for analysis of plaque morphology. The plaques were stained with crystal violet at 5 dpi (**B**). The sizes of plaques were measured and plotted (**C**). (**D**) BHK-21 cells were mock-infected or infected with SD12-F120 or SD12 and harvested for western blot analysis of NS1 and NS1ʹ expression with antibodies specific to NS1 and NS1’. Data are presented as mean ± SD from three independent experiments. * *p* < 0.05; ** *p* < 0.01; *** *p* < 0.001; **** *p* < 0.0001 tested by Student’s *t*-test.

The susceptible BHK-21 cells are frequently used to examine the virologic properties of the SA14-14-2 vaccine strain in vitro [26,27,28], so we therefore compared the replication kinetics of the attenuated SD12-F120 strain with its virulent parental SD12 strain in BHK-21 cells. BHK-21 cells were inoculated with SD12-F120 or SD12 and harvested at the indicated time points for the detection of JEV titers by plaque assay (Figure 1a). The viral titers of SD12-F120 in the supernatants peaked at 36 h post-infection (hpi) with the maximal titer of 10^7.57^ PFU/mL, while SD12 reached the maximal titer of 10^6.5^ PFU/mL at 48 hpi. This observation demonstrated that the attenuated SD12-F120 strain replicated more efficiently than its virulent parental SD12 strain in BHK-21 cells. Analysis of the plaque morphology formed on BHK-21 cells indicated that the plaque size of SD12 was 4.5 ± 0.8 mm which was significantly larger than that (3.5 ± 0.5 mm) of SD12-F120 (Figure 1b,c), showing a small-plaque property that has been regarded as an attenuation marker of SA14-14-2 vaccine strain [26].

An RNA pseudoknot-mediated ribosomal frameshift event occurring between codons 8 and 9 of JEV NS2A results in synthetization of a derivative NS1 (NS1’), in which a 52 amino acid peptide is added to the C-terminus of NS1 [35]. Ablating NS1’ expression contributes to the attenuation of SA14-14-2 strain [36], but does not impair the virulence of JaTH-IC strain [37]. We therefore detected whether SD12-F120 and SD12 produced NS1’ protein in BHK-21 cells by western blot analysis. As shown in Figure 1d, NS1’ together with NS1 was detectable in cells infected with SD12-F120 and SD12 with similar expression levels, suggesting that both strains expressed NS1’ protein in BHK-21 cells.

### 3.2. Reduced Replication Efficiency of the Attenuated SD12-F120 Strain in Mouse Brains and Mouse Primary Neuron Cells

**Figure 2:**
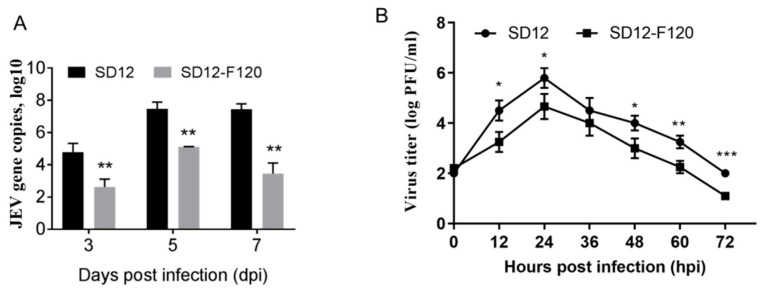
Replication of SD12-F120 and SD12 in mouse brains and mouse primary neuron cells. (**A**) Mice (10 mice/group) were intracerebrally inoculated with SD12-F120 or SD12. Brain samples were collected at 3, 5, and 7 dpi. The viral loads in the brain samples were measured by qRT-PCR. (**B**) Primary mouse neuron cells were infected with SD12-F120 or SD12 at an MOI of 0.01. The viral titers in the supernatants were measured by plaque assays. Data are presented as mean ± SD from three independent experiments. * *p* < 0.05; ** *p* < 0.01; *** *p* < 0.001 tested by Student’s *t*-test.

The attenuated JEV strains including SA14-14-2 vaccine strain [27] and SCYA201201-0901 strain [38] are able to replicate in mouse brain after intracerebral inoculation despite their attenuated phenotype. Given the highly attenuated phenotype of SD12-F120, we compared the replication efficiency of SD12-F120 with SD12 in mouse brains. Mice were intracerebrally inoculated with SD12-F120 or SD12 at a dose of 10^3^ PFU and brain samples were collected for analysis of the levels of JEV gene expression by qRT-PCR. JEV gene expression of both strains was detectable in the inoculated mouse brains at 3 dpi, peaking at 5 dpi, and then declined at 7dpi. However, the expression levels of SD12-F120 gene were significantly lower than those of SD12 at all days post-infection (Figure 2a), showing a reduced replication efficiency of the attenuated SD12-F120 strain in the nervous system compared with its virulent parental SD12 strain.

To confirm the reduced replication efficiency of SD12-F120 in the nervous system, we examined the replication kinetics of SD12-F120 and SD12 in mouse primary neuron cells. Mouse primary neuron cells were infected with SD12-F120 or SD12 and the viral titers in the supernatants were measured by plaque assay. As showed in Figure 2b, the mouse primary neuron cells have supported the replication of both strains, but the viral titer of SD12-F120 was lower than that of SD12, showing the reduced replication efficiency of the attenuated SD12-F120 strain in the mouse primary neuron cells compared with its virulent parental SD12 strain.

### 3.3. Differences in Neuroinvasiveness and Neurovirulence between the Attenuated SD12-F120 and Its Virulent Parental SD12 Strains in Mice

**Figure 3:**
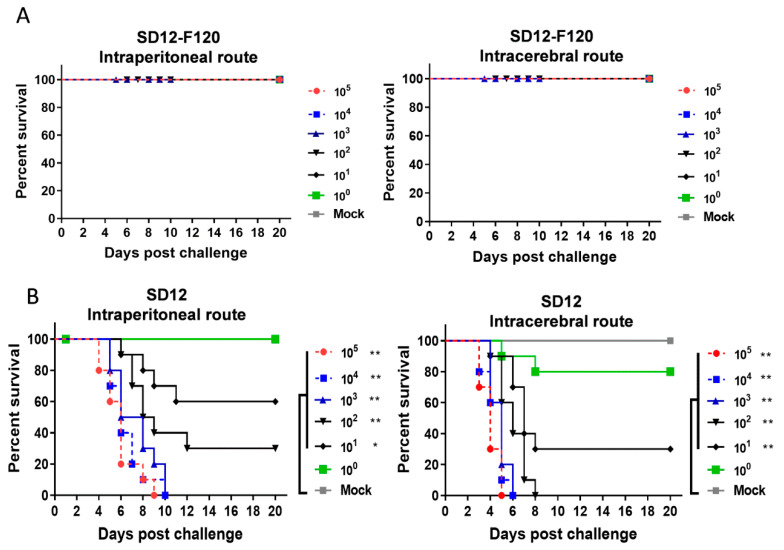
Neuroinvasiveness and neurovirulence tests in mice. Mice (10 mice/group) were inoculated intraperitoneally or intracerebrally with either SD12-F120 (**A**) or SD12 (**B**) at doses ranging from 10^0^ to 10^6^ PFU to measure neuroinvasiveness or neurovirulence, respectively. The mice were monitored daily for 20 days. Kaplan–Meier test was used for survival analysis. Asterisks indicate values that are statistically significant (* *p* < 0.05; ** *p* < 0.01).

Three-week-old weanling mice are a well-established small animal model for evaluation of JEV virulence including the neuroinvasiveness and neurovirulence [28]. We therefore inoculated the weanling mice intraperitoneally or intracerebrally with SD12-F120 and SD12 for testing their neuroinvasiveness or neurovirulence, respectively. The mice inoculated with SD12-F120 at doses ranging from 10^0^–10^6^ PFU via either intraperitoneal route or intracerebral route showed no clinical signs of JEV infection, even at the highest dose of 10^6^ PFU (Figure 3a). The estimated LD_50_ of neuroinvasiveness and neurovirulence for SD12-F120 was >10^6^ PFU. In contrast, the mice inoculated with SD12 via intraperitoneal route developed the clinical signs of JEV infection beginning from a dose of 10^1^ PFU. The LD_50_ of neuroinvasiveness for SD12 was 500 PFU (Figure 3b). Additionally, the mice inoculated with SD12 via intracerebral route produced the clinical signs of JEV infection starting from a dose of 1 PFU. The LD_50_ of neurovirulence for SD12 was 2.5 PFU (Figure 3b). Altogether, these data indicate that the SD12-F120 strain lost the neuroinvasiveness and neurovirulence in mice, showing a highly attenuated phenotype of virulence, whereas the virulent parental SD12 strain remained highly neuroinvasive and neurovirulant in mice, consistent with our previous results [30].

### 3.4. Protective Efficacy of the Attenuated SD12-F120 Strain against Its Virulent Parental SD12 Challenge Strain in Mice

**Figure 4:**
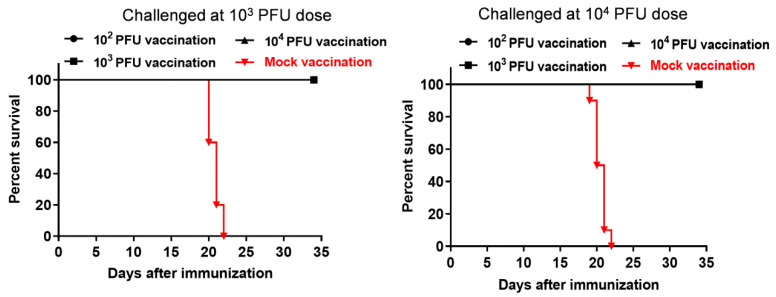
Protective efficacy of SD12-F120 against SD12 challenge in mice. Mice (8 mice/group) were mock-vaccinated or vaccinated intraperitoneally with SD12-F120 at doses ranging from 10^2^ to 10^4^ PFU. After 14 days of vaccination, the mice were challenged intraperitoneally with SD12 at a dose of 10^3^ and 10^4^ PFU, respectively, and were monitored daily for 20 days.

To determine whether the attenuated SD12-F120 could be used as a vaccine candidate strain, we examined the protective efficacy of SD12-F120 using a mouse challenge model [19]. Mice were immunized with SD12-F120 at doses ranging from 10^2^ to 10^4^ PFU and challenged after 14 days with SD12 at a dose of 10^3^ and 10^4^ PFU, respectively. All mock-vaccinated mice developed the clinical signs of JEV infection following the SD12 challenge and were sacrificed (Figure 4). In contrast, the mice vaccinated with SD12-F120 at doses ranging from 10^2^ to 10^4^ PFU survived without visible clinical signs, showing that attenuated SD12-F120 strain provided the vaccinated mice complete protection from the lethal dose challenge of its virulent parental SD12 strain.

### 3.5. Overview of Nucleotide and Amino Acid Variations between the Attenuated SD12-F120 and Its Virulent Parental SD12 Strains

**Figure 5:**
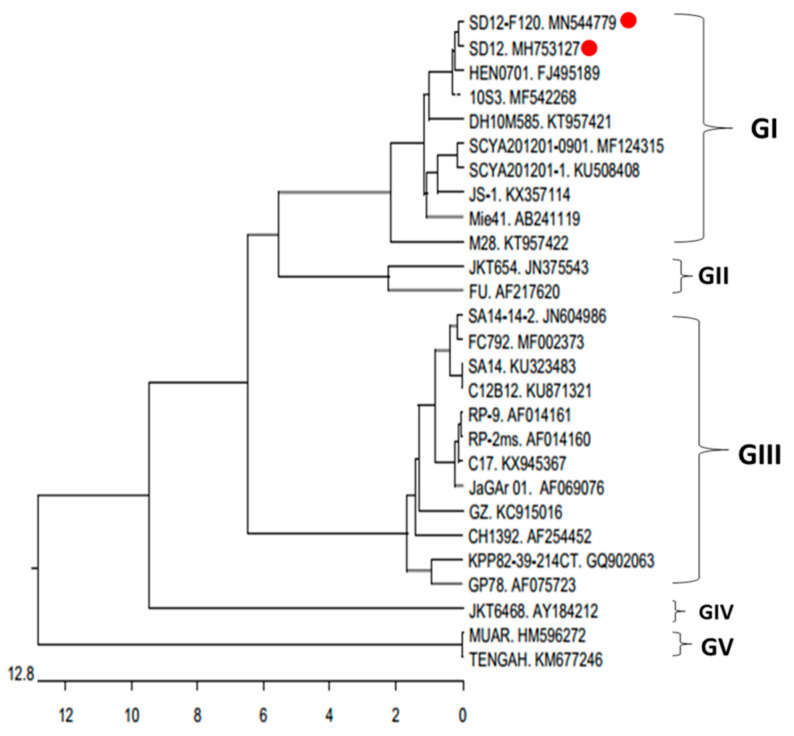
Phylogenetic tree of JEV strains based on the nucleotide sequence of JEV E gene. The phylogenetic tree was constructed by the neighbor-joining method using MEGA version 7.0 software. SD12-F120 and SD12 are labeled with red dot.

The genome sequence of SD12-F120 was determined and compared with SD12 sequence for analysis of their genetic variations. The resulting sequence of SD12-F120 was deposited in GenBank (GenBank No. MN544779). Phylogenetic analysis on the nucleotide sequence of JEV E gene revealed that SD12-F120 vs. SD12 pair was mostly closed to a GI isogenic pair of the attenuated 10S3 and its virulent parental HEN0701 strains originally isolated from aborted pig fetuses in China in 2007 [31] (Figure 5).

To determine the genetic changes acquired during the attenuation process, we compared the nucleotide and amino acid sequences between SD12-F120 and SD12. As shown in Table 1, a total of 29 nucleotide changes were observed in the virus genome, of which 20 changes were considered silent nucleotide mutations, while nine resulted in eight amino acid substitutions: two in E, one in NS1, two in NS3, one in NS4B and two in NS5 proteins. Notably, E^138^ residue of E protein that is considered to be associated with the attenuation of GI 10S3 strain [31] was changed from an acidic glutamic acid (E) to a basic arginine (R) in SD12-F120. E^176^ residue of E protein that is involved in the determination of SA14 neurovirulence [29,39] was substituted from I to T in SD12-F120.

### 3.6. Comparison of Amino Acid Substitutions in E Protein among Five Isogenic Attenuated and Virulent Strain Pairs

Amino acid substitutions in E protein are considered to be responsible for JEV attenuation/virulence [27,29,31]. To determine common amino acid substitutions in the E protein acquired during the attenuation process, we compared the amino acid variations of SD12-F120 vs. SD12 with those from other four isogenic pairs of the attenuated and their virulent parental strains: two from GI virus (SCYA201201-0901 vs. SCYA201201 pair [38], and 10S3 vs. HEN0701 pair [31]) and two from GIII virus (SA14-14-2 vs. SA14 pair [28], and RP-2ms vs. RP-9 pair [40]). The attenuation history of these isogenic pairs is listed in Supplementary Table S1. As shown in Table 2, each isogenic strain pair contained variable numbers of amino acid substitutions in the E protein with the highest numbers (9) of SA14-14-2 vs. SA14 pair and the lowest numbers (1) of 10S3 vs. HEN0701 and RP-2ms vs. RP-9 pairs. The substitution at E^138^ that has been demonstrated to be the key determinant of SA14-14-2 neurovirulence [29] was present in four out of five pairs, with the exception of SCYA201201-0901 vs. SCYA201201 pair. The substitution at E^176^ that is involved in the determination of SA14 neurovirulence [29,39] occurred in three pairs with the exception of 10S3 vs. HEN0701 and RP-2ms vs. RP-9 pairs. The remaining substitutions were specific to their respective strain pairs.

### 3.7. Comparison of Variations in Other Regions among Five Isogenic Attenuated and Virulent Strain Pairs

In addition to the amino acid substitutions of E the protein, the nucleotide and/or amino acid substitutions in other regions are also involved in the determination of JEV attenuation/virulence [41,42,43]. We first compared the amino acid variations in other viral proteins of SD12-F120 vs. SD12 pair with those from four other isogenic pairs of the attenuated and their virulent parental strains. As shown in Table 3, each isogenic strain pair contained variable numbers of amino acid substitutions in viral proteins with the highest numbers (10) in the SCYA201201-0901 vs. SCYA201201 pair and the lowest numbers (1) in the RP-2ms vs. RP-9 pair. Most of the variations were unique to their respective strain pair, with the exception of NS2B^65^, NS3^105^, NS5^277^ substitutions that were observed in two strain pairs. It is known that 5’-UTR is involved in the attenuation of SA14-14-2 strain [44], so we therefore compared the nucleotide variations in the UTRs among the five isogenic pairs of the attenuated and their virulent parental strains. All nucleotide variations were strain pair specific. No common substitutions were observed (Table 4).

## 4. Discussion

We compared the phenotypic and genotypic characteristics of the attenuated SD12-F120 strain with its virulent parental SD12 strain. SD12-F120 formed smaller plaque on BHK-21 cells and showed reduced replication in the mouse primary neuron cells and mouse brains compared with SD12. Mice inoculated with SD12-F120 up to 10^6^ PFU via either intraperitoneal or intracerebral route showed no clinical signs of JEV infection, indicating high attenuation in terms of both neuroinvasiveness and neurovirulence, while SD12 exhibited high neuroinvasiveness and neurovirulence in mice with LD_50s_ of 500 PFU and 2.5 PFU respectively. Mice immunized with SD12-F120 survived from the lethal dose challenge of SD12, showing a complete protective efficacy of SD12-F120. These attenuated phenotypic characteristics of SD12-F120 were mostly similar to those of SA14-14-2 vaccine strain [28,45].

SD12-F120 showed the smaller plaque phenotype; however, no reduced or delayed replication was observed in BHK-21 cells compared with its virulent parental SD12. This result was inconsistent with its replication kinetics in the mouse primary neuron cells as well as with a previous observation that SA14-14-2 vaccine strain shows a smaller plaque phenotype associated with slower replication kinetics in BHK cells as compared with its parental SA14 [28]. A possible explanation for this inconsistency was that the cell-specific adaptation beneficial to virus fitness on BHK-21 cells occurs following 120 passages on BHK-21 cells. However, the mechanism(s) responsible for the reduced plaque phenotype of SD12-F120 in BHK-21 cells needs to be examined by comprehensive experiments using a reverse genetic system of JEV.

Mice are highly susceptible to JEV challenge via either intraperitoneal or intracerebral routes and are well-established small animal model to study the virulence of JEV [28]. We observed that mice inoculated with SD12-F120 up to 10^6^ PFU via either intraperitoneal or intracerebral route showed no clinical symptoms, while mice inoculated with SD12 produced the clinical signs of JEV infection starting at 100 PFU via intracerebral route. These results indicated that SD12-F120 has a highly attenuated phenotype in terms of both neuroinvasiveness and neurovirulence. It is known that the phenotype of JEV virulence in mice is determined by viral proteins and UTRs [41,42,43], especially, the E protein that has been considered the main determinant of JEV virulence [29].

JEV E protein mediates receptor-mediated endocytosis and low pH-triggered membrane fusion [46] and plays a critical role in determination of JEV attenuation/virulence [29,41,47,48]. In this study, we observed two amino acid substitutions at E^138^ (E138R) and E^176^ (I176T) in the E protein of SD12-F120 compared with SD12. Residue E^138^ is positioned in the hinge region at the interface of domains I and II of E protein and the mutation at this point results in the changes in SA14-14-2 virulence [29,49] and therefore has been considered as the key determinant of SA14-14-2 neurovirulence [29]. In addition, the substitution of E^138^ from E to alkaline residues contributes to the attenuation of neurovirulence, while E-to-acidic residue replacement at E^138^ enhances the neurovirulence of 10S3/HEN0701 strain [31]. In the present study, E residue at E^138^ was replaced with an alkaline residue R in SD12-F120, suggesting that this mutation might contribute to attenuation. Residue E^176^ is located in the domain I that is involved in structural rearrangement and membrane fusion [38]. Substitution at E^176^ from I to R reduces the neurovirulence of SA14 strain in mice [39]. Revertant mutation at E^176^ from V to I slightly increases the neurovirulence of SA14-14-2 strain [29]. We observed the substitution at E^176^ (I176T) in SD12-F120, which might contribute to SD12-F120 attenuation. However, the significance of E^138^ (E138R) and E^176^ (I176T) substitutions in SD12-F120 attenuation needs to be examined by comprehensive experiments using a reverse genetic system of JEV.

In addition to E protein, other viral proteins are also involved in the determination of JEV attenuation/virulence [41,42,43]. We observed several substitutions in NS1, NS3, NS4B, and NS5 proteins of the attenuated SD12-F120 strain. One substitution (G177E) was present in NS1 that is a multifunctional glycoprotein with central roles in viral replication, eliciting the immune response, and inhibition of the complement system [50,51]. Two substitutions (L104P and V527G) were observed in the serine protease domain (L104P) and nucleoside 5’-triphosphatase domains (V527G) of NS3 that possesses the enzymatic activities of serine protease, helicase and nucleoside 5’-triphosphatase and is involved in the processing of the viral precursor polyprotein and the replication of viral genomic RNA [52,53]. One substitution (T46A) was shown in NS4B that plays an important role in viral replication by facilitating the formation of the viral replication complexes and in counteracting innate immune responses [54]. Two substitutions (A3P and E276K) were detected in NS5 that consists of the methyltransferase and RNA-dependent RNA polymerase and plays a critical role in JEV replication and inhibition of interferon response [55,56]. These substitutions may alter their roles in viral replication, immune modulation, and/or immune evasion, and thereby synergistically influence JEV attenuation/virulence. However, this needs to be defined by comprehensive experiments.

In addition to amino acid substitution, SD12-F120 harbored 20 silent nucleotide mutations. It is known that the substitution within UTRs and silent nucleotide mutation in the coding region may cause a variation of RNA secondary structures, regulatory RNA elements, and/or sub-genomic RNAs, thereby playing important roles in viral replication and host fitness. For example, the 5’-UTR of West Nile virus that is a member of JEV serocomplex within the genus *Flavivirus* of the family *Flaviviridae* [57] is important for RNA synthesis and virus replication [58], while a single silent nucleotide G66A mutation in NS2A gene of SA14-14-2 disturbs viral RNA replication in mice brains [36]. The contribution of these silent nucleotide mutations of SD12-F120 in the attenuation phenotype needs to be explored in the future.

To determine common amino acid substitutions that may be involved in JEV attenuation, we compared the amino acid variations of SD12-F120 vs. SD12 pair with those from other four isogenic pairs of the attenuated and their virulent parental strains and observed that the numbers and positions of amino acid variations were different from each other among the five pairs, showing a strain pair specific pattern. SA14-14-2 vs. SA14 pair harbored the most variations in numbers accounting for 17 (9 in E and 8 in other proteins), followed by SCYA201201-0901 vs. SCYA201201 pair (4 in E and 10 in other proteins), SD12-F120 vs. SD12 pair (2 in E and 6 in other proteins), 10S3 vs. HEN0701 pair (1 in E and 6 in other proteins), and RP-2ms vs. RP-9 pair (1 in E and 1 in other proteins). These differences in the numbers and positions of amino acid variations were probably attributable to the difference in the virus strain, passage history as well as the cell types used for passage. Notably, out of the five pairs, substitution at E^138^ position was identified in four pairs (SD12-F120 vs. SD12, 10S3 vs. HEN0701, SA14-14-2 vs. SA14, and RP-2ms vs. RP-9), suggesting that the residue at E^138^ position is likely to be the common genetic determinant responsible for JEV attenuation/virulence. However, revertant mutation of E^138^ modifies the neurovirulence only but not the neuroinvasiveness of SA14-14-2 strain, indicating that the residue at E^138^ position plays the most important role in sustaining neurovirulence, but not the attenuated neuroinvasiveness phenotype associated with SA14-14-2 strain [29]. In addition, no mutation at E^138^ is observed in SCYA201201-0901 vs. SCYA201201 pair, suggesting that mutations at other positions (E^176^, E^251^, and E^273^) other than E^138^ may contribute to the attenuated neurovirulence phenotype of SCYA201201-0901 strain [38,39]. In addition to E^138^, substitution at E^176^ position was observed in three pairs (SD12-F120 vs. SD12, SCYA201201-0901 vs. SCYA201201, and SA14-14-2 vs. SA14) out of the five pairs, suggesting that the residue at E^176^ position may also contribute to JEV attenuation/virulence. Substitution at E^176^ from I to R reduces the neurovirulence of SA14 strain in mice [39]. Revertant mutations at E^176^ from V to I slightly increase the neurovirulence of SA14-14-2 strain [29]. Besides the substitutions at E^138^ and E^176^ positions, most of the remaining amino acid variations were unique to their respective strain pairs and their roles in the determination of JEV attenuation/virulence remained unknown. Overall, our data together with the previous observations suggested that the genetic changes acquired during the attenuation process were likely to be strain-specific, implying the complexity of the mechanisms associated with JEV attenuation/virulence.

Analysis of the protective efficacy of the attenuated SD12-F120 indicated that SD12-F120 provided the vaccinated mice complete protection from the lethal dose challenge of its virulent parental SD12 strain. It is known that JEV E and prM proteins are able to induce a range of protective immune responses including JEV-specific B cells and cytotoxic T cells in mice and pigs [18]. Comparison of the amino acid sequence between SD12-F120 and SD12 revealed that the amino acid substitutions were present in E protein, but not in prM protein. JEV E protein has been considered the major immunogen capable of eliciting protective immunity since JEV-neutralizing antibodies specific to E protein alone are sufficient to confer protection against JEV challenge [18]. The neutralizing epitopes on E protein that is structurally divided into domains I, II, and III are mapped at the domain I-II hinge, domain I lateral ridge, domain III lateral ridge, and fusion loop. The residues critical for binding by JEV-neutralizing antibodies include E^104^, E^106^, and E^107^ located at the fusion loop, E^52^, E^126^, E^136^, and E^275^ at domain I-II hinge, E^179^ at domain I lateral ridge, E^302^, E^337^, E^360^, and E^387^ at domain III lateral ridge [46]. Interestingly, these residues critical for JEV neutralizing antigen were identical between SD12-F120 and SD12, which might play an essential role in the determination of the protective efficacy of SD12-F120. However, the mechanism(s) underlying the protective efficacy of SD12-F120 against SD12 challenge needs to be examined by comprehensive experiments using a reverse genetic system of JEV.

In conclusion, we compared the phenotypic and genotypic characteristics of SD12-F120 with SD12. SD12-F120 was highly attenuated in both neuroinvasiveness and neurovirulence in mice as compared with its virulent parental SD12. This isogenic pair could be used for exploring the mechanisms associated with JEV attenuation/virulence. The numbers and positions of amino acid variations between the attenuated and the virulent parental strains were different from each other among five isogenic pairs, suggesting that the genetic changes acquired during the attenuation process were likely to be strain specific. These findings could be useful for understanding the determination of JEV attenuation/virulence as well as for the development of GI vaccines. In addition, although SD12-F120 provided the immunized mice complete protection from SD12 challenge, more comprehensive experiments including examination of the genetic stability, attenuation stability, attenuation phenotypic stability as well as the protective efficacy against heterologous strain challenge should be conducted to evaluate the potential of SD12-F120 as a vaccine candidate.

## Figures and Tables

**Table 1:** Nucleotide and amino acid variations between SD12-F120 and SD12.

Region	Position		Nucleotide Changes		Amino Acid Changes		Amino Acid Substitutions
	Nucleotide	Amino Acid	SD12-F120	SD12	SD12-F120	SD12	
PrM	540	--*	T	C	--	--	--
E	1338	--	G	T	--	--	--
	1390 + 1391	138	AG	GA	R	E	E138R
	1505	176	C	T	T	I	I176T
	2055	--	C	T	--	--	--
	2460	--	A	G	--	--	--
NS1	2694	--	A	G	--	--	--
	3011	177	A	G	E	G	G177E
	3132	--	G	A	--	--	--
NS2A	3924	--	C	T	--	--	--
NS2B	4503	--	G	A	--	--	--
	4513	--	C	T	--	--	--
NS3	4922	104	C	T	P	L	L104P
	4974	--	A	G	--	--	--
	6006	--	T	C	--	--	--
	6191	527	G	T	G	V	V527G
NS4A	6726	--	T	C	--	--	--
	6735	--	T	C	--	--	--
NS4B	7407	46	C	T	A	T	T46A
NS5	7688	3	G	C	P	A	A3P
	8238	--	C	T	--	--	--
	8439	--	C	T	--	--	--
	8506	276	A	G	K	E	E276K
	8571	--	A	C	--	--	--
	8715	--	T	C	--	--	--
	9810	--	T	C	--	--	--
	9840	--	T	C	--	--	--
	10005	--	T	C	--	--	--

* Silent mutation.

**Table 2:** Amino acid substitutions in E protein between attenuated and virulent parental strains.

Position	Genotype I						Genotype III			
	Isogenic Strain Pair		Isogenic Strain Pair		Isogenic Strain Pair		Isogenic Strain Pair		Isogenic Strain Pair	
	Attenuated SD12-F120	Virulent SD12	Attenuated SCYA201201-0901	Virulent SCYA201201	Attenuated 10S3	Virulent HEN0701	Attenuated SA14-14-2	Virulent SA14	Attenuated RP-2ms	Virulent RP-9
72	A	A	T*	**A**	A	A	A	A	A	A
107	L	L	L	L	L	L	**F**	**L**	L	L
138	**R**	**E**	E	E	**R**	**E**	**K**	**E**	**K**	**E**
176	**T**	**I**	**R**	**I**	I	I	**G**	**I**	I	I
177	T	T	T	T	T	T	**A**	**T**	T	T
244	E	E	E	E	E	E	**G**	**E**	E	E
251	S	S	**Y**	**S**	S	S	S	S	S	S
264	Q	Q	Q	Q	Q	Q	**H**	**Q**	Q	Q
273	E	E	**K**	**E**	E	E	E	E	E	E
279	K	K	K	K	K	K	**M**	**K**	K	K
315	A	A	A	A	A	A	**V**	**A**	A	A
439	K	K	K	K	K	K	**R**	**K**	K	K
Total number of substitution	2		4		1		9		1	

* Substitutions are highlighted in red.

**Table 3:** Amino acid substitutions in other viral proteins between attenuated and virulent strains.

Protein	Position	Genotype I						Genotype III			
		Isogenic Strain Pair		Isogenic Strain Pair		Isogenic Strain Pair		Isogenic Strain Pair		Isogenic Strain Pair	
		Attenuated SD12-F120	Virulent SD12	Attenuated SCYA201201-0901	Virulent SCYA201201	Attenuated 10S3	Virulent HEN0701	Attenuated SA14-14-2	Virulent SA14	Attenuated RP-2ms	Virulent RP-9
C	66	L	L	L	L	L	L	**S**	**L**	L	L
prM	86	H	H	R*	**H**	H	H	H	H	H	H
	109	E	E	**D**	**E**	E	E	E	E	E	E
NS1	100	A	A	**T**	**A**	A	A	A	A	A	A
	177	**E**	**G**	E	E	E	E	E	E	E	E
	237	E	E	E	E	**G**	**E**	E	E	E	E
	269	D	D	**D**	**N**	D	D	D	D	D	D
	351	D	D	D	D	D	D	**H**	**D**	D	D
NS2B	44	V	V	**A**	**V**	V	V	V	V	V	V
	63	E	E	E	E	E	E	**D**	**E**	E	E
	65	E	E	E	E	E	E	**G**	**D**	**E**	**D**
NS3	59	M	M	M	M	M	M	**V**	**M**	M	M
	102	P	P	P	P	**P**	**Q**	P	P	P	P
	105	**P**	**L**	P	P	P	P	**G**	**A**	A	A
	527	**G**	**V**	G	G	G	G	G	G	G	G
NS4A	63	E	E	E	E	**D**	**E**	E	E	E	E
	96	A	A	**T**	**A**	A	A	A	A	A	A
	168	G	G	**E**	**G**	G	G	G	G	G	G
	248	F	F	**L**	**F**	F	F	F	F	F	F
NS4B	46	**A**	**T**	T	T	T	T	T	T	T	T
	106	I	I	I	I	I	I	**V**	**I**	I	I
	192	V	V	V	V	**A**	**V**	V	V	V	V
NS5	3	**P**	**A**	P	P	P	P	P	P	P	P
	277	**K**	**E**	E	E	**E**	**K**	E	E	E	E
	313	V	V	V	V	**A**	**V**	V	V	V	V
	386	H	H	H	H	H	H	**Y**	**H**	Y	Y
	623	V	V	**I**	**V**	V	V	V	V	V	V
	879	I	I	**T**	**I**	I	I	I	I	I	I
Total number of substitutions		6		10		6		8		1	

* Substitutions are highlighted in red.

**Table 4:** Nucleotide variations in the UTR regions between attenuated and virulent strains.

Region	Position	Genotype I						Genotype III			
		Isogenic Strain Pair		Isogenic Strain Pair		Isogenic Strain Pair		Isogenic Strain Pair		Isogenic Strain Pair	
		Attenuated SD12-120	Virulent SD12-01	Attenuated SCYA201201-0901	Virulent SCYA201201	Attenuated 10S3	Virulent HEN0701	Attenuated SA14-14-2	Virulent SA14	Attenuated RP-2ms	Virulent RP-9
5′-UTR	39	U	U	U	U	U	U	A*	**U**	U	U
	43	A	A	**G**	**A**	A	A	A	A	A	A
	52	G	G	**A**	**G**	G	G	G	G	G	G
3′-UTR	10408	C	C	**U**	**C**	--	C	--	--	--	--
	10428	U	U	U	U	U	U	C	U	U	U
	10679	C	C	**U**	**C**	C	C	C	C	C	C
	10705	C	C	C	C	**T**	**C**	C	C	C	C
	10731	A	A	**G**	**A**	A	A	A	A	A	A
	10960	G	G	G	G	A	G	G	G	G	G
Total number of substitutions		0		5		2		1		0	

* Substitutions are highlighted in red. -- Sequence not available.

## Supplementary Materials

The following are available online at https://www.mdpi.com/1999-4915/12/5/552/s1, Table S1: Attenuation history of JEV isogenic pairs, Table S2: Primers sequences used in this study.
