# Supplementary material for: Phenotypic and Genotypic Comparison of a Live-Attenuated Genotype I Japanese Encephalitis Virus SD12-F120 Strain with Its Virulent Parental SD12 Strain
Source: Viruses. 2020 May 16;12(5):552. doi: 10.3390/v12050552 (PMC7290960; doi:10.3390/v12050552)
Supplement: Supplementary file 1 [file viruses-12-00552-s001.pdf]

Supplementary Table 1 Attenuation history of JEV isogenic pairs

Isogenic strain pair	Attenuation history	Designated name	GenBank No.	Reference
SD12-F120 vs SD12 pair	Parental SD12 isolated from brain of an aborted swine fetus	SD12	MH753127	N/A
	100 serial passages of SD12 in BHK-21 cells, followed by three plaque purifications from small plaque variant on BHK-21 cells and virulence tests in three-week-old mice.	SD12-F100	N/A	
	120 serial passages in BHK-21 cells, followed by three plaque purifications on BHK-21 cells and virulence tests in three-week-old mice.	SD12-F120	MN544779	
SCYA201201-0901 vs SCYA201201 pair	Parental JEV SCYA201201-1 virus isolated from the brain tissues of aborted piglet.	SCYA201201-1	KU508408	Zhou Y, et al., 2018
	16 passages of SCYA201201-1 in BHK-21 cells.	SCYA201201	N/A	
	86 passages in BHK-21 cells and 3 plaque purifications of small plaque variant of SCYA2012-1.	SCYA201201-86	N/A	
	120 passages in BHK-21 cells and 3 plaque purifications with virulence selections of 3-week-old mice inoculated intracranially.	SCYA201201-0901	MF124315	
10S3 vs HEN0701 pair	Virulent HEN0701 is originally isolated from aborted pig fetus.	HEN0701	FJ495189	Zheng X, et al., 2018

	Derived from HEN0701 by 100 passages in BHK-21cells.	10S3	MF542268	
SA14-14-2 vs SA14 pair	Parental JE SA14 virus isolated from a pool of *Culex pipiens* larvae by 11 passages in mouse brain.	SA14	KU323483	Yun SI, et al., 2016
	100 serial passages in PHK cells, followed by three plaque Purifications.	SA14-12-1-7	N/A	
	Five suckling mice skin passages (s.c. inoculation) followed by two plaque purifications.	SA14-14-2	JN604986	
RP-2ms vs RP-9 pair	Virulent RP-9 is generated from a Taiwanese isolate NT109 using γ-ray irradiation.	RP-9	AF014161	Lin YL, et al., 1996
	Attenuated RP-2ms is also generated from a Taiwanese isolate NT109 using γ-ray irradiation.	RP-2ms	AF014160	

Supplementary Table 2 Primers sequences used in this study

	Primer	Genomic Position	Sequence 5′-3′
qRT-PCR	JEV probe	215-240	CGTGCAGTATGGTCGCTGCACACGGA^{FAM}
	JEV-F	119-138	CATGTGTGARGACACYATCA
	JEV-R	308-327	CCAGCCAAGCCTCTTTTTTR
Genome sequencing	JEV-1f	1-26	AGAAGTTTATCTGTGTGAACTTCTTG
	JEV-1r	2892-2913	ATCAGGGCATTCCTTTGTCTCG
	JEV-2f	2371-2391	GGGGCCTTACTTCTTTGGATG
	JEV-2r	5655-5675	GCATAGTCCGTGATCCATTCG
	JEV-3f	5455-5477	GTGATGGATGAGGCTCACTTCAC
	JEV-3r	9184-9204	CACCCCACCTCCTGAATTCTC
	JEV-4f	8791-8813	GTCAAGGAAGTGCTCAACGAGAC
	JEV-4r	10931-10954	TCTTCCTCACCACCAGCTACATAC
